# 

**Published:** 1838-01-01

**Authors:** 


### MEDICO-CHIRURGICAL REVIEW,

AND

### JOURNAL

OF

### PRACTICAL MEDICINE.

*(NEW SERIES.)*

VOLUME TWENTY-EIGHT,

[1st of OCTOBER, 1837, to 31st of MARCH,]

1838.

VOL. VIII. of DECENNIAL SERIES.

EDITED

By JAMES JOHNSON, M.D.

PHYSICIAN EXTRAORDINARY TO THE LATE KING,

AND

HENRY JAMES JOHNSON, Esq.

LECTURER ON ANATOMY AT THE SCHOOL OF ST. GEORGE'S HOSPITAL IN KINNERTON STREET.

Surgeon Genl's Office LIBRARY 57468 Brown

LONDON:

PUBLISHED BY S. HIGHLEY, 32, FLEET STREET,

*Re-printed in New York, by Mr. Wood.*

1838.

W1
ME75T

PRINTED BY F. HAYDEN,
Little College Street, Westminster.

### BIBLIOGRAPHICAL RECORD.

1. An Essay on Pyrexia, or Symptomatic Fever, as illustrative of the Nature of Fever in general. By Henry Clutterbuck, M.D. 8vo, pp. 136. Highley, Sept. 1837.

2. Observations on the Structure and Functions of the Spinal Cord. By R. D. Grainger, Lecturer on Anatomy, &c. &c. Octavo. Highley, 1837.

3. The Philosophy of Health; or, Exposition of the Physical and Mental Constitution of Man, &c. &c. By Southwood Smith, M.D. Volume the Second. Charles Knight. 1837.

4. A Lecture on the Nature and Cultivation of the Medical Profession. By Geo. Morgan, A.M., of Aberdeen. Highley, 1837.

5. Guy's Hospital Reports. No. 5, Oct. 1837. Edited by Dr. Barlow and Dr. Babington. Highley, 1837.

6. The Edinburgh Dissector; or System of Practical Anatomy, for the Use of Students in the Dissecting-room. By a Fellow of the College of Surgeons in Edinburgh. 8vo, price 9s. Baillière, London; Richard, Edinburgh, 1837.

7. Case of Hydrosis, or Hydrotic Fever: with Remarks. By John C. Lever, M.R.C.S. Octavo, pp. 24. London, Oct. 1837.

8. Elements of Anatomy. By Jones Quain, M.D. Fourth edition, revised and enlarged. Illustrated with Steel Plates and numerous Engravings on Wood. Part I. Price 12s. The Second and concluding Part will be ready in November, price 10s. Taylor and Walton, London. Oct. 1837.

9. A Treatise on the Malformations, Injuries, and Diseases of the Rectum and Anus. Illustrated with Plates. By Geo. Bushe, M.D. Formerly Professor of Anatomy and Physiology, &c. Octavo, pp. 299. New York, 1837.

10. Plates illustrating a Treatise on the Malformations, Injuries, and Diseases of the Rectum and Anus. By George Bushe, M.D. Quarto, New York, 1837.

11. Elements of Chemistry, including the recent Discoveries and Doctrines of the Science. By the late Edward Turner, M.D. Sixth Edition, enlarged and revised by Professor Leibez and Wilton G. Turner. Part I., price 7s. The Second Part will be published in November. London, Taylor and Walton, Oct. 1837.

12. Memoranda of difficult Subjects in Anatomy and Surgery; being a Pocket

Companion for Students freparing for the College of Surgeons. By Robt. Druitt, M.R.C.S. Price 2s. Renshaw, Oct. 1837.

☞ *Multum in parvo.*

---

13. A Treatise on the Influenza of 1837, containing an Analysis of One Hundred Cases observed at Birmingham. By Peyton Blakeston, M.A. Med. Licen. Cant. Physician to the Birmingham Dispensary. Octavo, pp. 60. Longman & Co. 1837.

---

14. A Discourse on some of the Diseases of the Knee-joint, delivered before the Massachuset's Medical Society, at their Annual Meeting, in May, 1837. By Geo. Hayward, M.D. Professor of Surgery, &c. in Harvard University. Octavo, pp. 28. Boston, 1837.

---

15. Observations on the Ganglionic Enlargement of the Pneumo-gastric Nerve, &c. By Mr. Ed. Cock. From the Guy's Hospital Reports.

---

16. A Series of Anatomical Plates, in Lithography, &c. By Jones Quain, M.D. and W. J. Erasmus Wilson. Division III. Nerves, III. Price Two Shillings. Fasciculus 52. Taylor and Walton. Oct. 1837.

---

17. Hints to Mothers, for the Management of Health during the Period of Pregnancy and in the Lying-in Room, &c. By Thomas Bull, M.D. &c. Duodecimo, pp. 174. Longman and Co. 1837.

---

18. A Conspectus of the Pharmacopœias, of the London, Edinburgh, and Dublin Colleges of Physicians; being a Practical Compendium of Materia Medica, and Pharmacy. By A. T. Thomson, M.D. F.L.S. &c. Tenth Edition, 1837.

☞ *This is an admirable pocket-book, worth its weight in gold.*

---

19. The Flora of Jamaica; a Description of the Plants of that Island, arranged according to the Natural Orders. With an Appendix, containing an Enumeration of the Genera, according to the Linnæan System, and an Essay on the Geographical Distribution of the Species. By James Macfadyen, M.D. Vol. I. Ranunculaceæ—Segummosceæ. Longman and Co. Nov. 1837.

---

20. Retrospective Address, delivered at the Fifth Anniversary Meeting of the Provincial Medical and Surgical Association, held at Cheltenham, July 19, 1837. By James L. Bardsley, M.D. Octavo, pp. 55. Longman and Co. 1837.

---

21. On Auscultation and Percussion in the Diagnosis of Diseases of the Organs of Respiration and Circulation, &c. By Julius Wolff, M.D., Member of the Royal College of Gottingen. 8vo, pp. 200. Highley, 1837.

---

22. Memoires de l'Academie Royale de Medecine. Tome Sixieme, quarto, avec Planches. Bailliere, Nov. 1837.

---

23. The History of British Birds. By William Yarrell, F.L.S. Part 3. Price 2s. 6d. London, 1837.

---

24. Elements of the Practice of Medicine. By Richard Bright, M.D., and Thomas Addison, M.D. Physicians to Guy's Hospital. Part the Second. Longman and Co. 1837.

---

25. A General View of the Structure of Animals, with its Adaptation to External Objects; being a Lecture delivered before the Birmingham Branch of the British and Foreign Young Men's Society, Sept. 12, 1837. By Langston Parker, M.R.C.S. &c. Octavo, sewed, pp. 28. Birmingham, Sept. 1837.

☞ *A very able address, evincing an intimate acquaintance with an extensive and interesting subject.*

---

26. A Treatise on the Diseases of the Larynx and Trachea, founded on the Essay to which was adjudged the Jacksonian Prize for 1835. Octavo, pp. 338. Longman's, Nov. 1837.

---

27. First Principles of Surgery, being an Outline of Inflammation and its Effects. By George T. Morgan, A.M., Lecturer on Surgery in Aberdeen. Part the *Second.* (First Part not received.) 1838. Price Five Shillings.

---

28. Prize Thesis: on the Presence of Air in the Organs of Circulation. By John Rose Cormack, M.D. Edinburgh, 1837. Octavo, pp. 55. Price 1s. 6d.

---

29. The Medical Pocket-book and Almanack for 1838. By John Foote, Jun. London, Renshaw, Nov. 1837.

---

30. A Treatise on Hernia; comprising the Surgical Anatomy, Operative Surgery, and Treatment of that important Disease, &c. By MALCOLM W. HILLES, Author of the "British Dissector." Octavo, pp. 52. Renshaw, London, Nov. 1837.

31. Practical Surgery. With One Hundred and Twenty Engravings on Wood. By ROBERT LISTON, Surgeon. Octavo, pp. 494. Churchill, London, Nov. 1837.

32. A Statistical Report of the Addenbrooke's Hospital, for the Year 1836.—From the Transactions of the Cambridge Philosophical Society. By HENRY JOHN H. BOND, M.D. Quarto, Cambridge, 1837.

33. A Treatise on Cow-pox, in which the Existence of Small-pox, or Varioloid in any Form, subsequent to Vaccination, is shewn to arise from some Imperfection in its Performance, &c. By DAVID R. HEBBARD, M.D. New York, 1835.

34. An Essay on the Classification of the Insane. By M. ALLEN, M.D. Octavo, pp. 212. With Plates. Taylor, London, Nov. 1837.

35. Institutes of Surgery; arranged in the Order of the Lectures delivered in the University of Edinburgh. By Sir CHARLES BELL, F.R.S. &c. Octavo, pp. 353. In two volumes. Vol. I. Black & Co. Edinb. Nov. 1837.

36. Changes produced in the Nervous System by Civilization, considered according to the Evidence of Physiology and the Philosophy of History. By ROBT. VERITY, M.D. Octavo, pp. 79. Highley, Nov. 1837.

37. The Transactions of the Provincial Medical and Surgical Association. Instituted, 1832. Part I. Vol. VI. Sherwood, Nov. 1837. With Plates.

38. Obstetric Plates, with Explanations, selected from the Anatomical Tables of WM. SMELLIE, M.D. Octavo, Highley, Nov. 1837.

☞ *These plates are twelve in number, judiciously selected, and ably executed. The whole in a portable form, and only five shillings in price.*

39. Address delivered before the Medical Society of the State of New York, Feb. 8th, 1837. By JAMES M'NAUGHTEN, M.D.

☞ *There is very considerable research in this Address, and great information respecting the state of medicine and medical institutions in America.*

40. Remarks on the Treatment of Fractures in the Lower Extremities, without the Aid of Splints. With Cases. By JOHN BURKE, M.R.C.S. Octavo, sewed. London, Highley.

41. On Diseases of the Rectum. By JAMES SYME, F.R.S. Octavo, pp. 138. Edinburgh, 1837.

42. Observations on Abdominal Tumors and Intumescence; illustrated by some Cases of Acephalocyst Hydatids. By Dr. BRIGHT. (From the Guy's Hospital Reports.)

43. Physical Education; or the Nurture and Management of Children, founded on the Study of their Nature and Constitution. By SAMUEL SMILES, Surgeon. Small 8vo. Oliver and Boyd, Edinburgh, Nov. 1837.

44. The Phrenological Journal, and Magazine of Moral Science. No. 54. Quarterly. Dec. 1st, 1837.

☞ *This is the first of a new series, and is published in London. We think the editors as well as the publishers are changed. This is certainly a good number.*

45. The Chirurgico-Comico-Hyeroglyfico. By PILL-BOX. Six Figures on Stone. Price One Shilling.

☞ *This is an amusing publication, and will be completed in 24 numbers, namely, a number for each letter.*

46. Electricity; its Nature, Operation, and Importance in the Phenomena of the Universe. By WILLIAM LEITHEAD, Secretary to the London Electrical Society. One vol. small 8vo.

☞ *This subject is treated in a manner at once to interest the general and the professional reader. Several medical subjects, as inflammation, fever, &c. are handled with considerable ingenuity.*

47. Observations on the Preservation of Health in Infancy, Youth, Manhood, and Age:—with the best Means of improving the Moral and Physical Condition of Man. By JOHN HARRISON, CURTIS, Esq. Small 8vo, pp. 128.

☞ *In this small volume Mr. Curtis has not attempted anything new; but only to render that which is well known to the scientific, familiar to the general reader.*

